# An Automated High-Throughput Cell-Based Multiplexed Flow Cytometry Assay to Identify Novel Compounds to Target *Candida albicans* Virulence-Related Proteins

**DOI:** 10.1371/journal.pone.0110354

**Published:** 2014-10-28

**Authors:** Stella M. Bernardo, Christopher P. Allen, Anna Waller, Susan M. Young, Tudor Oprea, Larry A. Sklar, Samuel A. Lee

**Affiliations:** 1 Section of Infectious Diseases, New Mexico Veterans Healthcare System, Albuquerque, NM, United States of America; 2 Division of Infectious Diseases, University of New Mexico Health Science Center, Albuquerque, NM, United States of America; 3 Center for Molecular Discovery, University of New Mexico, Albuquerque, NM, United States of America; University of Wisconsin Medical School, United States of America

## Abstract

Although three major classes of systemic antifungal agents are clinically available, each is characterized by important limitations. Thus, there has been considerable ongoing effort to develop novel and repurposed agents for the therapy of invasive fungal infections. In an effort to address these needs, we developed a novel high-throughput, multiplexed screening method that utilizes small molecules to probe candidate drug targets in the opportunistic fungal pathogen *Candida albicans*. This method is amenable to high-throughput automated screening and is based upon detection of changes in GFP levels of individually tagged target proteins. We first selected four GFP-tagged membrane-bound proteins associated with virulence or antifungal drug resistance in *C. albicans*. We demonstrated proof-of-principle that modulation of fluorescence intensity can be used to assay the expression of specific GFP-tagged target proteins to inhibitors (and inducers), and this change is measurable within the HyperCyt automated flow cytometry sampling system. Next, we generated a multiplex of differentially color-coded *C. albicans* strains bearing C-terminal GFP-tags of each gene encoding candidate drug targets incubated in the presence of small molecules from the Prestwick Chemical Library in 384-well microtiter plate format. Following incubation, cells were sampled through the HyperCyt system and modulation of protein levels, as indicated by changes in GFP-levels of each strain, was used to identify compounds of interest. The hit rate for both inducers and inhibitors identified in the primary screen did not exceed 1% of the total number of compounds in the small-molecule library that was probed, as would be expected from a robust target-specific, high-throughput screening campaign. Secondary assays for virulence characteristics based on null mutant strains were then used to further validate specificity. In all, this study presents a method for the identification and verification of new antifungal drugs targeted to fungal virulence proteins using *C. albicans* as a model fungal pathogen.

## Introduction

*Candida* infections are a major cause of morbidity and mortality in a wide range of patient populations. *Candida albicans* is a normal human commensal organism, although if host defense mechanisms are compromised, invasive candidiasis can occur in virtually any host. *Candida* species are now the fourth most common cause of hospital-acquired bloodstream infections, and the third most common cause of nosocomial urinary tract infections [1]–[3]. Despite improvements in antifungal therapy, the high attributable mortality rate due to *Candida* infections has improved little from two decades ago. Current antifungal agents are limited in the following aspects: (i) available formulations, (ii) accessibility to the site of infection and ability to achieve therapeutic dose, (iii) hepato- and nephro-toxicity, and/or (iv) emergence of antifungal drug resistant strains [4], [5]. Azole antifungals, although usually well-tolerated, are limited by substantial resistance in several important non-albicans *Candida* species and within *C. albicans* biofilms. Amphotericin B, including lipid-formulations, while broadly effective against many fungal pathogens, is limited by substantial toxicities [6]. The echinocandins, are costly, and while highly effective against most *Candida* species, can be ineffective in the presence of point mutations in the *FKS1* gene which encodes the glucan synthase drug target [7], [8]. Although the overall clinical prevalence for echinocandin-resistant *Candida* species remains low to date, there are increasing reports of echinocandin-resistant clinical isolates, particularly with the revision of Clinical Laboratory and Standards Institute (CLSI) clinical interpretative breakpoints for echinocandin susceptibilities [9]–[12]. Furthermore, echinocandins have limited central nervous system and urinary penetration, and are of limited to no utility against *Cryptococcus* species, some other rare opportunistic yeast species, and many invasive mold infections [13], [14].

As a result of the continually recurring problem of drug resistance and antimicrobial therapy, efforts have turned towards developing therapeutic methods and preventative measures that target virulence-specific aspects of a microorganism rather than its growth. This approach is attractive because it can potentially circumvent the development of drug resistant isolates, as it targets the microorganism’s potential to become virulent rather than directly targeting its growth. More importantly for antifungal drug development, there is currently a lack of fungal-specific targets, and virulence factors would thus serve as unique targets [15]. Research groups in the field have identified a growing number of *Candida albicans* virulence-related proteins, and many of these are cell surface, cell membrane-associated, or secreted proteins, including the secreted aspartyl proteases (Saps), which are degradative enzymes thought to assist in adherence and tissue invasion [16],[17]. The ability to switch between yeast and hyphal morphology and/or to form biofilms has also received considerable attention [15], [18], [19] and there has been substantial recent effort to discover drugs against *Candida* biofilms including the development of a high-throughput *Candida* biofilm chip assay [20]. However, despite the identification and characterization of a number of potential drug targets, development of novel antifungal drugs has been lacking. Targeting of virulence factors has been an area of intensive study, particularly against bacterial pathogens. There are a number of promising anti-virulence compounds in pre-clinical development as anti-bacterial agents, although this strategy is still in its early stages [21]. This approach requires a detailed understanding of molecular pathogenesis. Importantly, significant resources are required for the pursuit of this strategy, with relatively fewer resources devoted to antifungal drug discovery. Thus, a concerted and collaborative effort between academic investigators, government and other funding sources, biotechnology companies, big pharma, and other stakeholders will be required to make substantial progress in the clinical development of anti-virulence compounds and vaccines for antifungal therapeutics [15]. Furthermore, new antifungal agents take a considerable amount of time in the developmental pipeline, including specific tests for safety-related issues. Thus, our objective was to design a method that would allow us to screen a small-compound drug library comprised of FDA-approved agents against specific proteins known to be required for antifungal resistance or virulence, in order to identify agents not previously known to have antifungal activity. The fact that the agents in this library are already FDA-approved, with long-term safety information already at hand, can potentially reduce the time and resources required to conduct clinical trials of the repurposed drug.

We first selected several membrane proteins associated with antifungal drug resistance or virulence in the opportunistic fungal pathogen *Candida albicans* as potential drug targets. *CDR1* encodes a multi-drug efflux pump that has been shown to be overexpressed in *C. albicans* laboratory and clinical isolates that are phenotypically resistant to fluconazole and other azole antifungal drugs [5], [22]–[24]. In the clinically important biofilm lifestyle of *C. albicans*, which leads to intrinsic fluconazole resistance, overexpression of *CDR1* occurs in the early stages of biofilm formation [25], [26]. *C. albicans FTR1* encodes a membrane protein required for iron transport [27], [28]. Deletion of this gene results in a *C. albicans* mutant strain that is defective in virulence in a standard mouse model of disseminated candidiasis. *MLT1* encodes a vacuolar ABC-type transporter, which is also required for wild-type virulence [29]. *C. albicans SUR7* encodes a membrane-related protein that marks sites of endocytosis in the plasma membrane [30], [31]. Absence of this gene results in defective biofilm formation, and in vitro macrophage killing [29], and attenuates virulence in the mouse model for disseminated candidiasis [32]. Thus, the membrane proteins encoded by these genes represent potential novel drug targets.

Next, we took advantage of the recent progress in flow cytometry and its applications for high-throughput screening to design a high-throughput screening approach [33], [34]. Specifically, we employed the techniques of multiplexing [35] and the HyperCyt sampling system and its associated software programs [36], [37], to increase the speed and efficiency by which data acquisition and analysis is performed. The technique of fluorescent cell barcoding, previously described by Krutzik and Nolan [35] increases the number of cell types that can be assayed in a single well by differentially staining each cell type with distinct fluorophores. Its use in a cell-based high-throughput screening assay was successfully demonstrated in the non-pathogenic yeast, *Saccharomyces cerevisiae*, in a study designed to identify novel inhibitors of the yeast TORC1 complex [38]. Further, we utilized the flow cytometry aspect of the screen to monitor protein levels of select *C. albicans* target proteins fused with the green fluorescent protein (GFP) at the C-terminal end, which maintains expression of each protein under control of the native promoter [39]. We reasoned that the fluorescence signals can be detected using a flow cytometer and act as a direct measure of protein levels within the live *C. albicans* cell. We thus developed a cell-based high-throughput multiplex screening assay to simultaneously probe several candidate drug targets in a single chemical library screen.

## Materials and Methods

### Strains and media

All strains are listed in Table 1. The strains carrying GFP-tagged virulence-related proteins are heterozygous for the *YFG-*GFP allele. Strains were routinely grown at 30°C in YPD [1% (w/v) yeast extract, 2% (w/v) peptone, 2% (w/v) glucose] or Complete Synthetic Medium (CSM) without uridine [0.67% (w/v) yeast nitrogen base without amino acids, 2% (w/v) glucose, 0.68% (w/v) complete synthetic mixture without uridine (MP Biochemicals, Solon, OH)]. Uridine was added at a final concentration of 80 µg ml^−1^ where required. Solid media was prepared by adding 2% (w/v) agar. For all high-throughput screening (HTS) assays, CSM was used as the base growth medium because YPD has fluorescence in the violet channel and confounds multiplex discrimination (data not shown). The final growth medium used for the HTS assays contained 0.015% (v/v) Pluronic F68 (Gibco, Life Technologies, Grand Island, NY). Iron-rich medium consisted of CSM supplemented with additional FeCl_3_ at a final concentration of 1000 µM. Iron-deplete medium was CSM depleted of Fe^3+^ ions through the addition of 200 µM bathophenanthrolinedisulfonic acid (Sigma, St. Louis, MO) [40].

**Table 1:** *Candida albicans* strains used in this study.

Strain Name	Parent	Genotype	Source
SC5314		wild-type	
DAY185		*ura3*Δ*::λimm434/ura3*Δ*::λimm434 his1::hisG/HIS1::his1::hisG arg4::hisG/ARG4::URA3::arg4::hisG*	[47]
BWP17		*ura3*Δ/*ura3*Δ *arg4*Δ/*arg4*Δ *his1*Δ/*his1*Δ	[48]
*CDR1*-GFP	BWP17	*ura3*Δ/*ura3*Δ *arg4*Δ/*arg4*Δ *his1*Δ/*his1*Δ *CDR1*/*CDR1*-*GFP*-*HIS1*	[42]
*FTR1*-GFP	BWP17	*ura3*Δ/*ura3*Δ *arg4*Δ/*arg4*Δ *his1*Δ/*his1*Δ *FTR1*/*FTR1*-*GFP*-*HIS1*	[42]
*MLT1*-GFP	BWP17	*ura3*Δ/*ura3*Δ *arg4*Δ/*arg4*Δ *his1*Δ/*his1*Δ *MLT1*/*MLT1*-*GFP*-*HIS1*	[42]
*SUR7*-GFP	BWP17	*ura3*Δ/*ura3*Δ *arg4*Δ/*arg4*Δ *his1*Δ/*his1*Δ *SUR7*/*SUR7*-*GFP*-*HIS1*	[30]

### Creating a multiplex

**Figure 1:**
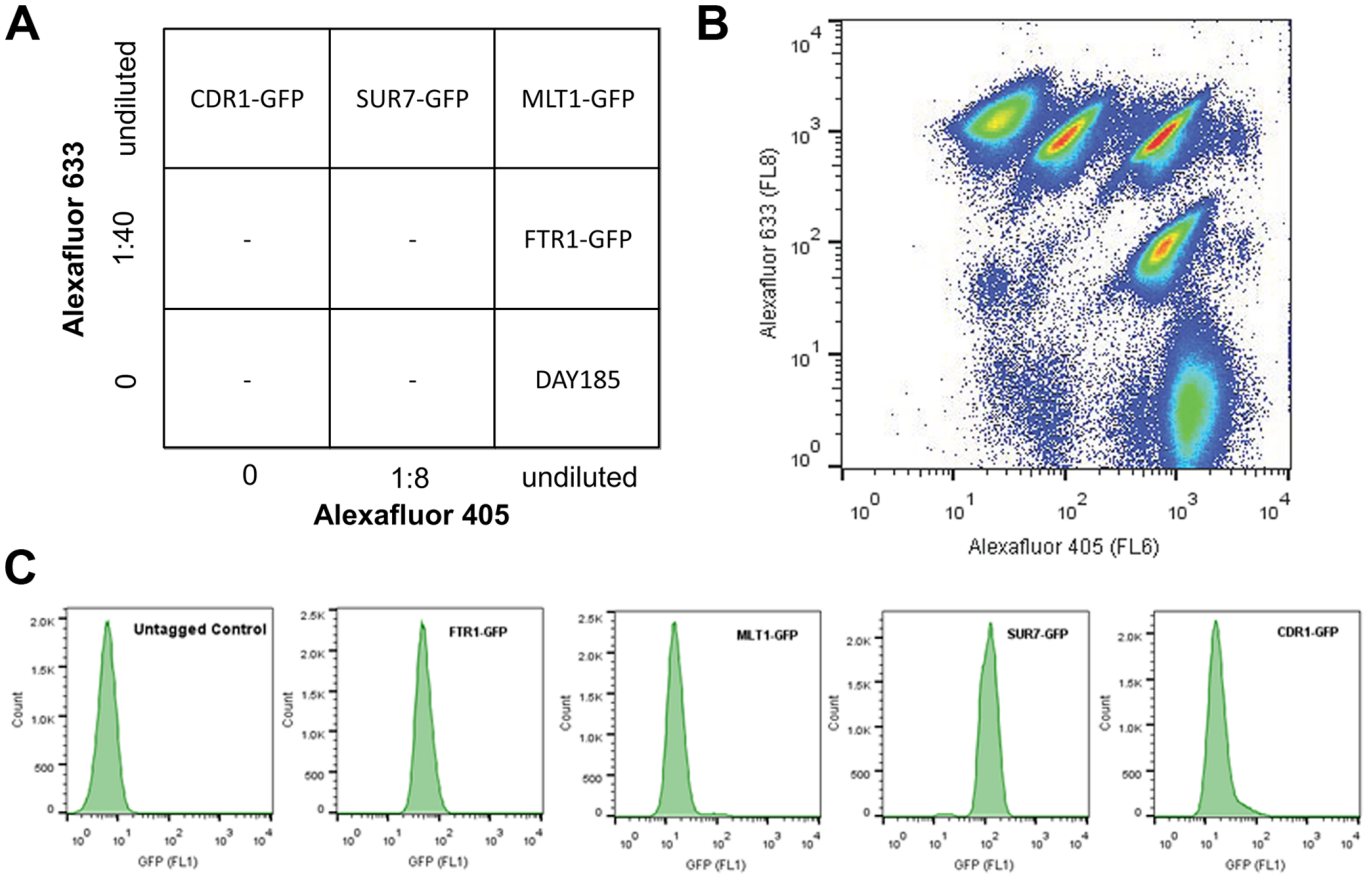
Multiplexing strategy to increase assay throughput. A schematic illustrating the amount of red and/or violet dyes used to differentially stain up to 9 different strains is shown in (A). Each sector of the 3×3 block represents one strain to be stained and details the specific concentration and combination of Alexafluor 633 and Alexafluor 405 dyes to be used for multiplexing. A typical sampling from one well carrying equal volumes of each stained cell is shown in a density plot in (B). Individual strains are visualized in a density plot of the two fluorescent channels (FL8 vs FL6), from which data on GFP levels can be extracted. Daughter cells that have lost some or all of the stain migrate to the lower left corner of the histogram. Gating on individual cell populations in the density plots depicted in (B) demonstrate that each population of cells is specific for one strain, with negligible contamination from migrating cells (C).

To increase the throughput of the HTS assay, each strain of interest was differentially color-coded with fluorescent dyes so that they can be combined and simultaneously interrogated in a single assay well (multiplex) [35]. The cell-based multiplex used in this study was generated using the five *C. albicans* strains of interest: CDR1-GFP, FTR1-GFP, MLT1-GFP, SUR7-GFP, and the untagged control strain, DAY185 (gift of A.P. Mitchell, Carnegie Mellon University). Differential staining was used to discriminate the different strains in the multiplex and was achieved by the addition of Alexafluor dye mixtures according to the diagram illustrated in Fig. 1. Thus, one milliliter cultures of each strain, grown as described in the Primary High-Throughput Screen (see below), were washed once with HHB buffer (30 mM HEPES, 110 mM NaCl, 10 mM KCl, 1 mM MgCl_2_ · 6H_2_O, 10 mM glucose, pH 7.4) and resuspended in HHB buffer containing 0.015% (v/v) Pluronic F68 (Gibco, Life Technologies, Grand Island, NY). Next, 10 microliters of dye mixture was added and the cells were incubated at 30°C for 30 minutes. Following this incubation, the cells were then washed in HHB buffer and resuspended in CSM. Equal volumes of each strain were combined and sampled through the HyperCyt system to check for successful dye incorporation and good separation (Fig. 1).

### Primary high-throughput screen

Overnight cultures of each strain, grown in YPD, were used to inoculate fresh CSM at a starting OD_600 nm_ of 0.2 which was grown to a final OD_600 nm_ of 0.5 at 30°C, followed by staining to create the multiplex. Following staining, the strains were combined and diluted into fresh media at 0.2 OD_600 nm_. 20 µL aliquots of the multiplex were transferred into 384-well plate format pre-populated with one library compound per well (columns 3 to 22) such that the final concentration of test compound was approximately 10 µM in growth medium using both Beckman Coulter Biomek NX MC and Span-8 liquid handling workstations (Beckman Coulter, Inc., Indianapolis, Indiana). The final concentration of DMSO in these wells was 1% (v/v). The first column of the plate was reserved for the negative or solvent-only control wells, wherein DMSO was added at a final concentration of 1% (v/v) in place of a test compound. The last two columns contained only growth medium and served as wash wells between row-to-row cell sampling to eliminate sample carry over. The second column would traditionally have been reserved for the positive control wells which would contain a known inhibitor of any one of the target proteins to be assayed. Currently, there are no known compounds that would inhibit expression of our proposed drug targets and thus the initial HTS assay was performed in the absence of a positive control compound, with the aim of identifying virulence-specific inhibitors. The assay plates were then incubated at 30°C for 2 hours using end-over-end rotation on a Hematology/Chemistry Mixer 346 (Fisher Scientific, Pittsburgh, PA). Following incubation at 30°C, the cells were diluted with Peptide Dilution Buffer (PDB) [0.1% (w/v) BSA, 1 mM magnesium chloride, 10 mM potassium chloride, 110 mM sodium chloride, 30 mM HEPES, pH 7.4] to achieve a final OD_600 nm_ of 0.2 prior to automated sampling of cells using the HyperCyt system [36], [37] connected to a Beckman Coulter Cyan ADP flow cytometer (Beckman Coulter, Inc., Indianapolis, Indiana), with light scatter and emission of violet (λ_Ex_ = 405 nm, λ_Em_ = 450 nm), green (λ_Ex_ = 488 nm, λ_Em_ = 530 nm), and red (λ_Ex_ = 635 nm, λ_Em_ = 665 nm) fluorescence data analyzed using HyperView software. Compounds that had innate violet, green, or red fluorescence were excluded by mathematical filters based on the compound’s ‘shifting’ impact on the lowest stained population. In total there were 5 green, 22 red, and 60 violet compounds, in the Prestwick Chemical Library. Identification of hits, either inducers or inhibitors, was based on constraints formulated from the Fold Response (the ratio between fluorescence measurement of sample well and fluorescence measurement of DMSO control for each strain). Compounds were considered hits according to the following criteria: A compound was classified as an inducer (inhibitor) if Fold Response was greater (lesser) than average plus (minus) three times the standard deviation of the particular strains’ fold response across entire compound library. Compounds that fell under the definition of a “hit” but elicited a response in the untagged control strain DAY185 were classified as non-specific and were thus excluded from analysis in secondary assays.

### Dose-response assay

Following the primary, high-throughput library screen, compounds that were identified as “hits” were assessed in a multiplexed, dose-response assay to verify target specificity. The cells and assay plates were prepared as for a primary screen but with drugs being serially diluted 3-fold across each row, starting at a concentration of 0.02 mg ml^−1^. The final concentration of DMSO in all wells, including those that did not receive a test compound, was 1% (v/v). Following a two-hour incubation at 30°C with end-over-end rotation, the cells were diluted with PDB and sampled through the HyperCyt system. Data were collected and analyzed as described for the Primary High-Throughput Screen.

### Statistical analysis

Zhang et al. [41] describe a statistical calculation, termed Z’-factor, that is used to determine if the difference observed between the positive and negative control is significant thus making a screen feasible. Z’-factor is calculated per the following equation:
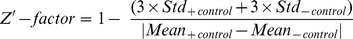



where Std is the standard deviation of the population, either positive control (+control) or negative (−control) and Mean is the average for those populations. Note the use of the absolute value of the differences between each Mean value. To compare the effect of a compound of interest (i.e., a “hit”) on a target, a similar equation can be applied:



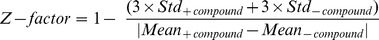
where data from when the compound of interest is present (+compound) or absent (−compound) are used.

### Secondary virulence-related assays

In order to validate specificity, virulence assays were performed to evaluate the effect of candidate compounds on the wild-type *C. albicans* strains SC5314 and DAY185. We had previously characterized the role of *C. albicans SUR7* in *Candida* virulence, specifically the result of loss-of-function of Sur7p [30], and thus selected a few potential inhibitors identified in our screen against strain SUR7-GFP. We performed characterization of wild-type strains in terms of lipase secretion, filamentation, biofilm formation, and virulence using an in vitro murine macrophage killing assay, as described previously [30], in the presence or absence of a potential inhibitor identified in the Primary High-Throughput Screen and Dose-response assays. The final concentration of DMSO, in which the compounds tested were dissolved, was 1% (w/v) in all assays performed.

## Results and Discussion

### Preliminary characterization of C. albicans strains encoding fluorescently-tagged membrane proteins

We developed a screening approach to assay the levels of *C. albicans* candidate drug targets by conjugating the protein of interest with green fluorescent protein (GFP), which can then be detected and quantified as the live cell carrying this fusion protein passes through the flow cytometer. In this study, we used a series of *C. albicans* strains in which GFP was fused to the C-termini of the plasma membrane transporters Cdr1p and Ftr1p, the vacuolar membrane ABC transporter Mlt1p, and the plasma membrane protein Sur7p [30], [42]. The fluorescence level of each GFP-tagged strain (indicated by the median channel fluorescence, MCF) was compared to the untagged control strain, DAY185, using the HyperCyt system [36], [37]. With respect to DAY185, the MCF of strains CDR1-GFP, FTR1-GFP, MLT1*-*GFP, and SUR7-GFP were on average 3-, 7-, 2-, and 18-fold higher, respectively (Table 2). These results show that GFP-tagged proteins of interest can readily be detected using the HyperCyt system and that the fluorescence of each strain is significantly higher than baseline fluorescence to allow for the detection of compounds that result in down-regulation of the GFP-tagged proteins. Due to the lack of known compounds that could act as positive controls for any of the strains, potential Z’-factors were calculated for each strain. The untagged control served as the positive control with the supposition that a compound that inhibits expression of each protein would result in fluorescence similar to the untagged control stain. The resulting calculations indicated that for three of the strains (SUR7-GFP, MLT1-GFP, and FTR1-GFP), high-throughput screens for inhibitors would be feasible (i.e., Z’-factor >0.5) (Table 2). The potential Z’-factor for strain CDR1-GFP suggests that this strain is not suitable for high-throughput screening, due to the relatively low MCF in combination with the resulting variability (standard deviation) in data generated from this strain (Table 2). This is in contrast to MLT1-GFP which yielded an acceptable potential Z’-factor of 0.5; despite having a relatively low MCF, the standard deviation of the data was very low.

**Table 2:** Measured green fluorescence (median channel fluorescence, MCF) of each strain used in the Primary Screen.

	*CDR1-GFP*	*SUR7-GFP*	*MLT1-GFP*	*FTR1-GFP*	DAY185 (untagged control)
MCF ± StdDev.	15.2±4.9	98.3±8.9	13.4±0.7	39.6±2.5	5.6±0.6
Relative MCF to control, DAY185	2.7x	17.5x	2.4x	7.0x	1x
Potential Z’-factor*	−0.73	0.69	0.50	0.72	

*Calculated with untagged control (DAY185) as the potential positive control for Inhibitor screens.

### Modulation and detection of the GFP-tagged protein target

**Figure 2:**
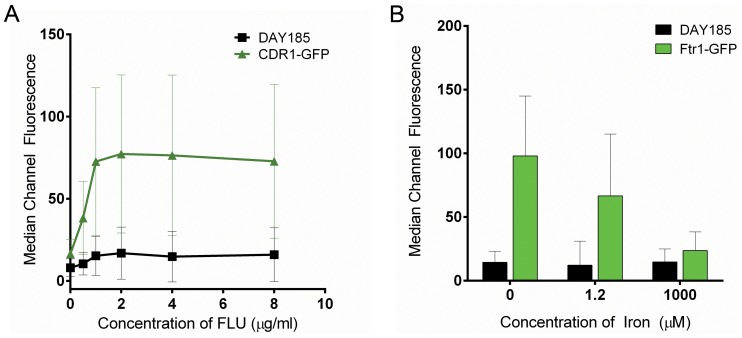
Changes in levels of GFP-tagged protein are readily detected. (A) Cells were grown in varying concentrations of fluconazole (FLU) and the response was measured using the HyperCyt system. Compared to the untagged control strain, a significant increase in fluorescence levels was detected for CDR1-GFP. Similarly, we queried the response of the FTR1-GFP strain to changes in iron content of the growth medium (B). These results demonstrate that we can detect changes to protein levels of our target proteins. Error bars indicate standard deviation values of the gated population of cells sampled and not of replicates of biological samples.

Next, in order to provide proof-of-principle that modulation of GFP fluorescent intensity could be used to assay response to a drug, we grew the strains CDR1-GFP and FTR1-GFP under conditions which have been previously shown to alter expression of the target protein. Expression of the multidrug efflux pump, Cdr1p, has been shown to increase in response to azoles [23], [43]. Thus, we queried the resulting GFP levels of the CDR1-GFP strain grown in varying concentrations of fluconazole, over time. In response to sub-inhibitory concentrations of fluconazole, a large increase in fluorescence occurred between 5 and 6 hours, and increased over time (with a maximal effect noted at 24 hours). A modest baseline increase in fluorescence was seen in the untagged control strain incubated with fluconazole, even after multiple washes, possibly due to intrinsic fluorescence of fluconazole. However, there is a much greater relative induction of CDR1-GFP expression, in identical conditions. At a fluconazole concentration of 0.002 mg ml^−1^, fluorescence is increased by 8.9 MCF (2.1-fold) in the untagged control strain, and by 60.8 MCF (4.9-fold) in the CDR1-GFP strain (Fig. 2A). Expression of *FTR1* mRNA is regulated by the amount of iron in the growth medium [27]. We thus grew strains FTR1-GFP and DAY185 in different media containing varying amounts of iron and measured the resulting changes in GFP levels using the HyperCyt system. We demonstrated that we could clearly detect compensatory increases (in low iron, or Fe-limiting conditions) and decreases (in high iron, or Fe-sufficient conditions) in Ftr1p-GFP expression, with the latter GFP-levels measuring close to the unlabeled control strain, DAY185, strongly suggesting complete depletion of the protein (Fig. 2B). The resulting changes in GFP levels are in agreement with the expected changes in mRNA levels in response to iron levels in the medium [27]. These results strongly suggest that modulation of *FTR1* by iron extends to the protein level, which to our knowledge has not been previously demonstrated. Taken together, these preliminary experiments demonstrate proof-of-principle that expression of GFP-tagged membrane proteins can be modulated by exposure to agents that can regulate protein expression and that these changes are readily detected and quantified using the HyperCyt system.

In this study, we expect to identify compounds that are able to modulate the levels of specific (target) proteins which have been previously been shown to be involved in *C. albicans* virulence. It should be noted that there could be many underlying mechanisms by which these compounds result in decreased levels of the proteins of interest, and that these compounds are not necessarily expected to interact with nor inhibit the target proteins directly. These specific mechanisms would be of great interest and would further our understanding of fungal virulence and pathogenesis. Whatever the specific mechanism may be, the objective is to identify compounds which decrease the levels of virulence-related proteins, thereby reducing virulence.

### Differential staining of the *C. albicans* strains to increase high-throughput readiness

To markedly increase the throughput efficiency of the assay, each strain of interest was differentially color-coded with fluorescent dyes so that they could be simultaneously be queried with a compound in a single well [35]. Each strain, including the untagged control strain, was labeled with different amounts of Alexafluor dyes, as indicated in Fig. 1A. All five distinct populations of cells were readily distinguishable (Fig. 1B) from which MCF data for green fluorescence could be extracted. Furthermore, the GFP histogram from each population of cells exhibited the expected distribution of GFP levels from a single-strain population indicating that unrelated signals from other strains were not captured within the gated dataset (Fig. 1C). The multiplex also eliminated any potential confounding effect of unstained daughter cells as they simply migrated to the lower left-hand corner of the density plot and were excluded from measurement by gating. After a two-hour incubation, the number of mother (stained) cells left was more than sufficient for data analyses (data not shown).

We also conducted single-plex experiments for each strain to examine changes in fluorescence between stained and unstained cells. There was no statistically significant difference in the ratio of median channel fluorescence to forward scatter (MCF/FSC ratio) (data not shown). Thus, although the median fluorescence was lower in the unstained population (likely due to the fact that they are new daughter cells with less surface area and therefore fewer membrane proteins), the relative fold change in GFP expression between daughter and parental cells was similar. Thus, any potential issue with unstained daughter cells during high-throughput screening is unlikely to be significant. Overall, these results demonstrate that multiplexing, by increasing the number of targets assayed per well, can greatly increase assay throughput.

### High-throughput screening of the Prestwick Chemical Library (PCL)

Having demonstrated that we can detect modulation of the expression of our target proteins (both up- and down-regulation) and further increase high-throughput readiness of the assay by multiplexing, we next screened the Prestwick Chemical Library (PCL) for compounds that could alter expression of our target proteins. We probed the PCL, which consisted of 880 off-patent drugs at the time of screening, in a 384-well multiplex format. In this initial screen, we identified agonists and/or antagonists of GFP expression in the CDR1-GFP, FTR1-GFP, MLT1-GFP, and SUR7-GFP strains, respectively (Table 3). Of note, this screening approach identified a number of compounds in the PCL that have been known to induce expression of Cdr1p, such as azoles and estrogen [44], [45] (Table 4). The relatively high hit rate for the number of inducers identified against Cdr1p is not surprising considering that the protein functions as a general efflux pump which protects against cell toxicity. Overall, the hit rate of the other target proteins did not exceed 1% of the total number of compounds tested, thus suggesting utility in screening much larger compound libraries.

**Table 3:** Number of initial hits identified in the PCL screen.

	CDR1-GFP	SUR7-GFP	MLT1-GFP	FTR1-GFP
Inducers	22	1	0	0
Inhibitors	0	10	5	9
Confirmed Hits	12 (out of 22 tested)	4 (out of 11 tested)	3 (out of 5 tested)	6 (out of 9 tested)

**Table 4:** List of PCL compounds confirmed in dose-response assays.

	CDR1-GFP		SUR7-GFP		MLT1-GFP		FTR1-GFP	
**Compound**	(**↑,↓**)*	**EC_50_**	(**↑,↓**)*	**EC_50_**	(**↑,↓**)*	**EC_50_**	(**↑,↓**)*	**EC_50_**
3-alpha-hydroxy-5-beta-androstan-17-one	↑							
Alexidine dihydrochloride			↓	12.1	↓		↓	7.4
Amiodarone hydrochloride	↑	n.d.					↓	n.d.
Androsterone	↑	26.3						
Beclomethasone dipropionate	↑	5.6						
Benzamil hydrochloride			↓					
Benzbromarone			↓	18.4	↓	16.0	↓	
Berlambine	↑	2.4						
Butamben	↑							
Capsaicin	↑							
Clopamide			↓					
Daunorubicin hydrochloride	↑	13.0						
Dyclonine hydrochloride	↑							
Epiandrosterone	↑							
Equilin	↑	6.8						
Estradiol-17 beta	↑	17.8						
Flufenamic acid			↓					
Haloprogin			↓	13.0	↓	9.1	↓	4.7
Hexamethonium dibromide dihydrate			↑					
Isoconazole	↑							
Lasalocid sodium salt	↑	8.6						
Mechlorethamine hydrochloride			↓				↓	6.8
Menadione							↓	
Methyl benzethonium chloride			↓		↓		↓	3.7
Miconazole	↑	23.9						
Mometasone furoate	↑	1.0						
Norethynodrel	↑							
Nystatine			↓				↓	24
Piperine	↑	27.7						
Sulconazole nitrate	↑	23.9						
Suloctidil	↑							
Testosterone propionate	↑	6.4						
Thimerosal			↓	6.9	↓	1.4	↓	0.5
Tomatidine	↑							

*The arrow indicates whether a compound elicited an increase (inducer) or decrease (inhibitor) in expression levels, as measured by fold-response of the GFP-tagged protein.

EC_50_ (in µM) values are reported for each respective compound confirmed as a hit, as determined in subsequent dose-response assays.

“n.d.” signifies that the hit was not confirmed in dose-response assays and therefore, no EC_50_ values have been determined.

We then cherry-picked 32 of the compounds for validation in dose-response multiplex assays utilizing the HyperCyt where cells were incubated with each compound of interest at varying concentrations. The number of compounds that confirmed activity in dose response assay with EC_50_ less than 30 µM are listed in Table 4, showing that across the strains the average percentage of false positives was 50%. Compounds that did not elicit a response were removed as false-positives. Moreover, using the calculation of Z-factors for each confirmed hit compound, we showed that the difference in GFP levels effected by each compound was statistically significant from baseline GFP levels of the target protein, thereby confirming these compounds as “hits” in the primary screen.

There are no known inhibitors of the candidate drug targets we pursued, and we anticipate that most, if not all, subsequent campaigns to identify targets of virulence will *a priori* lack appropriate inhibitors for use as a positive control in the screen. While the initial screen of the PCL was performed without a positive control, initial hits are verified using dose-response assays performed in exactly the same manner as the HTS library screen, and Z-factor calculations from the highest, triplicate concentration data points indicate that the effect of each compound tested is statistically significant (Z-factor >0.5). Thus, following assessment of specificity, any one of these hits can be used as positive controls for inhibitors (or inducers) of protein expression in subsequent screens of larger chemical libraries.

### Secondary phenotypic assays to evaluate virulence specific to the target

The primary goal of the screen was to identify agents that could be used to target virulence as opposed to those that directly affect cell growth. The cell-based assay that we describe identifies potential compounds that specifically alter the expression of proteins involved in virulence, without necessarily targeting the protein directly. The proteins we present here are candidate drug targets based on previous phenotypic characterizations undertaken with classical, reverse-genetic approaches wherein modulation of protein levels have resulted in altered virulence or virulence-related characteristics of the organism. Thus we used secondary assays designed to validate the hits that have passed verification in dose-response assays. In theory, if the compound of interest decreases the levels of the protein of interest, addition of the antagonistic compound will result in a wild-type *C. albicans* strain that will exhibit the characteristics of a heterozygous (if haploinsufficiency exists) or homozygous null mutant strain.

**Figure 3:**
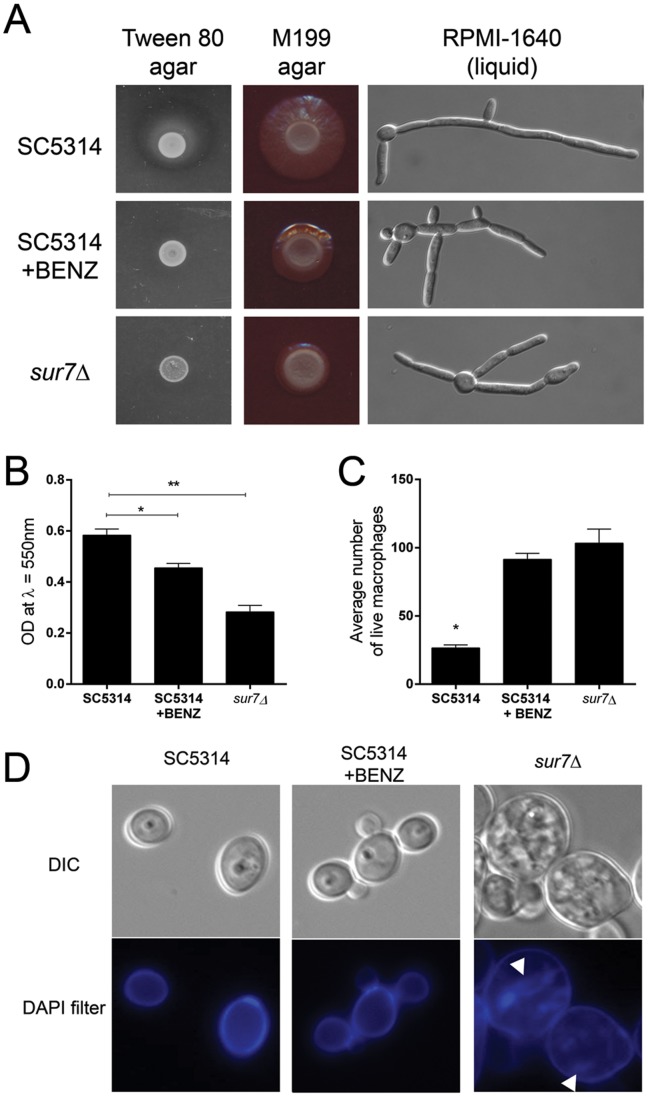
Secondary microbiologic and virulence assays further eliminate false-positive hits. To further eliminate false-positive hits, the effects of benzbromarone (BENZ, at a concentration of 18.4 µM) on wild-type *C. albicans* were tested and the results were compared to the phenotypic characteristics of the *sur7*Δ null mutant strain. In most cases, in the presence of benzbromarone, wild-type strains SC5314 and DAY185 mimicked the phenotypic profile of the *sur7*Δ mutant strain. Only results for SC5314 are shown in each panel. (A) The defects in secretion of degradative lipases, as indicated by the lack of the zone of precipitation surrounding the colony grown on Tween 80 agar, and in the inability to form mature hyphae on filamentation-inducing media (M199 agar and RPMI-1640) suggest that benzbromarone may act to reduce levels of Sur7p. (B) The ability of benzbromarone to inhibit biofilm formation of wild-type *C. albicans* was also tested and compared to the defective biofilm formed by the *sur7*Δ null mutant. Since metabolic activity can vary between strains of *C. albicans*, the resulting biofilm mass was measured using the crystal violet staining method [49]. When grown in the presence of benzbromarone, biofilm formation was reduced compared to wild-type growth. However, the reduction in biofilm formation of the benzbromarone-treated cells was not comparable to the biofilm formed by the *sur7*Δ strain. Statistical significance is indicated with asterisks, with p<0.0001. (C) The ability to kill macrophages by *C. albicans* was assessed by co-incubation of wild-type yeast cells with murine macrophage cells, in the presence or absence of benzbromarone. Virulent, wild-type *C. albicans* kill a significant proportion of macrophage cells when co-incubated for a period of 24 hours. When benzbromarone is present, a significant number of macrophage cells survive the co-incubation with wild-type *C. albicans*, similar to the *sur7*Δ null mutant strain. Statistical significance is indicated with an asterisk, with p<0.0001. (D) Cell wall morphology of the yeast form was characterized by staining yeast cells grown in the absence or presence of benzbromarone. Loss of function of *SUR7* results in plasma membrane invaginations and distinct cell wall-derived structures within the cell, which stain with calcofluor white as highlighted with arrows [30], [31]. At the concentration of benzbromarone tested, the distribution and staining of cell wall material in wild-type cells grown in the presence of benzbromarone was indistinguishable from wild-type grown cells.

Here, we present our analysis of a potential inhibitor affecting expression of *C. albicans* Sur7p. Benzbromarone (BENZ), a known uricosuric drug used to treat gout, which was one of 10 compounds that elicited a decrease in GFP levels from the SUR7-GFP strain. The Z-factor calculated from the dose-response assay was 0.91, indicating a statistically significant response of Sur7p to benzbromarone. Previous characterization of the role of *C. albicans SUR7* in pathogenesis demonstrated that the *C. albicans* null mutant strain is defective in a number of virulence-related phenotypes such as lipase secretion, filamentation, and biofilm formation [30]. Hence we tested wild-type strains DAY185 and SC5314 in these phenotypic assays, in the presence and absence of benzbromarone. Benzbromarone inhibited lipase secretion and filamentation of the wild-type strains to the same degree as that exhibited by the homozygous null mutant strain, *sur7*Δ (Fig. 3A). Likewise, biofilm formation is also inhibited in the presence of benzbromarone (Fig. 3B). We next assayed the effect of benzbromarone on the ability of wild-type *C. albicans* to kill macrophage in an in vitro assay for virulence utilizing murine macrophage. In this assay, we have previously shown that the *sur7*Δ homozygous null mutant is hypovirulent compared to the wild-type control strain, DAY185 [30]. In the presence of benzbromarone, both wild-type strains tested were hypovirulent compared to controls wherein benzbromarone was omitted (Fig. 3C). The resulting attenuation in virulence in the presence of benzbromarone was similar to levels exhibited by the *sur7*Δ mutant strain. ANOVA indicate that the difference between these conditions are statistically significant (p<0.0001). These preliminary results would seem to suggest that benzbromarone may affect Sur7p levels, thus resulting in homozygous null-like phenotypes. However, upon closer investigation of the cellular morphology of wild-type strains grown in the presence of benzbromarone, we did not observe the expected morphological defects in the plasma membrane that is characteristic of the *sur7*Δ homozygous null mutant (Fig. 3D). The latter phenotypic characterization suggests that the effect of benzbromarone is not limited to the reduced levels of Sur7p.

It is clear that the decrease in GFP levels detected by the flow cytometer do not necessarily translate to a wild-type strain that, when assayed in the presence of the inhibitory compound, exhibits reduced virulence similar to that of the corresponding homozygous null mutant. This is due to the many possible mechanisms by which a compound may effect a reduction in target protein levels. This caveat further stresses the importance of the secondary phenotypic virulence-related characterizations that follow verification of each identified hit, as part of the workflow in the identification of lead compounds that target virulence.

### Identification of compounds with general antifungal activity

While the immediate goal of the method described here is to identify novel compounds that can be directed against proteins involved in virulence, either when used alone or in conjunction with standard therapy, we realize that it can also identify novel and re-purposed compounds with general, non-specific antifungal properties as well. Rather than be discarded, further detailed characterization of compounds with general antifungal properties, but not virulence-specific activity, would still be undertaken as part of a high-throughput screening workflow. To demonstrate the potential of non-specific compounds identified in our screen of the PCL, we recently evaluated flufenamic acid for its potential use as an antifungal agent [46]. High doses of flufenamic acid were effective for both the prevention of biofilm formation, and inhibition of mature *C. albicans* biofilms. More importantly, low dose flufenamic acid (8 µg ml^−1^), when used in combination with standard antifungal agents, increased the potency of the latter in preventing the formation of *C. albicans* biofilms [46]. These results stress the importance of further studies on compounds that, although having been set aside in the initial high-throughput screen, may possess general antifungal activity.

## Conclusions

We developed a novel, cell-based, high-throughput screening method that can be used for the identification of compounds that alter protein expression in the model opportunistic fungal pathogen *C. albicans*. In this method, the screen starts with the thoughtful identification of potential targets based on previous characterization of loss-of-function and/or gain-of-function mutations in the genes (encoding the target proteins) of the microorganism of interest based on detailed studies of microbial pathogenesis. With the completion of genome sequences of important and clinically relevant microorganisms, we have seen a dramatic increase of information on protein function, among which are potential virulence-specific drug targets. It would appear that a major gap remains in the ability to translate this information to clinically relevant applications. With the growing interest in targeting virulence in light of the clinical failures of current antimicrobial agents and well-publicized development of multiple drug-resistant “super-bugs”, the method presented here represents a potential means to further such translational studies. This highly efficient, automated high-throughput screening method serves as a means to identify compounds that may be (i) general antifungal agents, (ii) used in virulence-targeted therapies, and/or (iii) inhibiting cellular pathways which, following mechanistic studies, can aid in the further understanding of pathogenesis of clinically relevant fungal pathogens.
